# Managing leprosy reactions with secukinumab: Insights from a case series

**DOI:** 10.1590/0037-8682-0118-2025

**Published:** 2025-09-29

**Authors:** Luciana Vilela Gomide, João Paulo Turri Brufatto, Thiago Jessé Kucarz, Paulo Eduardo Neves Ferreira Velho, Andréa Fernandes Eloy da Costa França

**Affiliations:** 1 Universidade Estadual de Campinas, Faculdade de Ciências Médicas, Campinas, SP, Brasil.; 2 Departamento de Dermatologia e Medicina Tropical, Instituto de Patologia Tropical e Saúde Pública, Universidade Federal de Goiás, Goiânia, GO, Brasil.

**Keywords:** Leprosy, Erythema nodosum leprosum, Leprosy reactions, Biological therapy

## Abstract

**Background:**

Erythema nodosum leprosum (ENL) is an immunological inflammatory reaction requiring precise management. Recalcitrant cases, their refractoriness to conventional therapy, and the adverse effects of standard treatments underline the need to explore alternative therapeutic options.

**Methods:**

We conducted a retrospective study of five patients treated with secukinumab between January 2023 and January 2025.

**Results:**

Patients showed clinical improvement with a reduced ENL frequency, neuritis relief, and corticosteroid sparing.

**Conclusions:**

Secukinumab appears to be a promising corticosteroid-sparing option for ENL, with a favorable safety profile. Further studies are required to confirm the efficacy and long-term safety of this treatment regimen.

Leprosy is a chronic infectious disease caused by *Mycobacterium leprae* and, less commonly, *M. lepromatosis*. Classified as a neglected tropical disease by the World Health Organization (WHO), leprosy remains a significant public health challenge, with 182,815 new cases reported in 184 countries in 2023^1^. It primarily affects the skin and peripheral nerves, potentially leading to severe disabilities if left untreated. Among its most debilitating complications are leprosy reactions, acute immunological episodes, which can cause substantial morbidity. These reactions significantly impair quality of life owing to neuritis, systemic symptoms, and painful skin lesions^2^.

Erythema nodosum leprosum (ENL) is a type 2 leprosy reaction that affects approximately 50% of multibacillary patients, particularly those with borderline and lepromatous forms². It is characterized by a type III hypersensitivity reaction involving immune complex deposition and neutrophil activation, leading to widespread inflammation. The roles of tumor necrosis factor-alpha (TNF-α), interleukin (IL)-6, and IL-17 in ENL pathogenesis have been increasingly recognized, highlighting new potential therapeutic targets^3^.

Standard ENL treatment typically involves systemic corticosteroids and/or thalidomide. However, long-term use of these medications is associated with significant adverse effects such as osteoporosis, diabetes mellitus, and thromboembolism^4^. Moreover, some patients do not respond adequately to these therapies^5^. Given these limitations, there is an urgent need for alternative treatments with fewer side effects and corticosteroid-sparing potential². Recent studies suggest that biological agents, particularly IL-17 inhibitors, are a promising therapeutic approach for managing ENL with a more favorable safety profile^6^ ^,^ ^7^.

We evaluated the efficacy and safety of secukinumab, an IL-17A inhibitor, for the management of ENL. We conducted a retrospective, single-center cohort study investigating the medical records of five males diagnosed with refractory ENL and treated with secukinumab. Patients were monitored at a tertiary care center in Brazil between January 2023 and January 2025.

Patients were included if they met the following criteria: (1) diagnosis of chronic ENL based on clinical presentation and histopathology, when available; (2) inadequate response to standard treatment, including systemic corticosteroids (prednisone ≥40 mg/day) and at least one other therapy (thalidomide, methotrexate, or mycophenolate mofetil); and (3) initiation of secukinumab because of poor response to standard treatment and/or severe ENL episodes.

Secukinumab (300 mg) was administered subcutaneously weekly for 4 weeks (induction phase), followed by a maintenance dose of 300 mg every 2 or 4 weeks, depending on the clinical response. Statistical comparisons were performed using a within-subject design, comparing each patient’s data before and after secukinumab initiation to assess the treatment effects while minimizing inter-individual variability. Paired t-tests were used for statistical analysis. The primary outcomes were ENL symptom control and reduced corticosteroid dependence. Secondary outcomes included alleviation of neuritis symptoms and improvement in overall quality of life.

All the patients were men, with a mean age of 35.6 years. Two had borderline leprosy, and three had a lepromatous form. Four patients completed 24 months of standard multidrug therapy (MDT) with rifampicin, dapsone, and clofazimine; one had completed 12 months. ENL developed during MDT in 80% of the cases (Table 1). Clinically, all patients exhibited nodular, edematous, infiltrative, or necrotic lesions, often accompanied by systemic symptoms, such as fever. Neuritis was present in all cases and predominantly affected the ulnar and fibular nerves.

**TABLE 1:** Clinical and therapeutic profile of patients with leprosy undergoing secukinumab treatment for the management of leprosy reactions.

Cases	Data of leprosy		Characteristics of ENL			Treatment of ENL			
	Age/Sex	Leprosy clinical type	ENL x MDT	ENL type	Neuritis	Previous treatment	Adverse events	Secukinumab dose (first dose)	Follow up (months)
**1**	45/M	LL	Started with MDT	Classic	Yes	Thalidomide	-	Attack + 300 mg/m	5
								(Aug/2024)	
						CS (oral*)			
						Pentoxifylline			
						Methotrexate			
**2**	26/M	BL	Started with MDT	Classic	Yes	Thalidomide	DVT	Attack + 300 mg/m	15
						CS (oral*/IV)	PE	(Oct/2023)	
						‘MMF	Obesity	300 mg/14 d	
							Diabetes	(April/2024)	
**3**	36/M	LL	Before MDT	Vasculonecrotic	Yes	Thalidomide CS (IV)	Osteoporosis	Attack + 300 mg/m	8
							Pathological fractures	(May/2024)	
							Obesity		
**4**	30/M	LL	After MDT	Classic	Yes	Thalidomide	Diabetes	Attack + 300 mg/m	8
						Methotrexate		(May/2024)	
**5**	41/M	BL	After MDT	Classic	Yes	Thalidomide	Obesity	Attack + 300 mg/m	9
								(April/2024)	

**LL:** lepromatous leprosy; **BL:** borderline leprosy; **ENL:** erythema nodosum leprosum; **MDT:** multidrug therapy; **CS:** corticosteroids (*prednisone, prednisolone, betamethasone); **IV:** intravenous; **MMF:** mycophenolate mofetil; **DVT:** deep vein thrombosis; **PE:** pulmonary embolism; **DM2:** type 2 diabetes mellitus. **Legend:** The table presents Information on the type of leprosy, type of leprosy reaction, completion of MDT, use of associated medications, initiation of secukinumab, sex, and age of patients included in the study.

Prior to secukinumab administration, all patients received systemic corticosteroids (prednisone) along with the non-steroidal anti-inflammatory drugs thalidomide (100-400 mg/day), methotrexate (15-25 mg/week), or mycophenolate mofetil (1.5 g/day). These therapies provided only partial ENL control and were associated with significant adverse effects, including deep vein thrombosis, pulmonary embolism, osteoporosis with pathological fractures, diabetes mellitus, and obesity (Figure 1).

**FIGURE 1:**
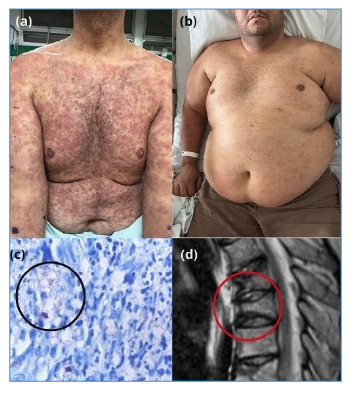
Corticosteroid adverse events. **(A):** Erythemato violaceous plaques, slightly ulcerated, at leprosy diagnosis with type 2 vasculonecrotic reaction (sweet-like). **(B):** Cushingoid patient after high doses of prednisone, with multiple fractures due to pathological osteoporosis. **(C):** Bacilli highlighted by Fite-Faraco staining (black circle) on histopathological examination. **(D):** CT scan showing a pathological vertebral fracture (red circle).

Following secukinumab treatment initiation, all patients showed significant clinical improvement. A marked reduction was noted in the frequency and severity of ENL episodes, resolution of systemic symptoms, such as fever and malaise, and improvement in neuritis symptoms, resulting in better functional outcomes. Notably, corticosteroid dependence decreased in all patients, with sustained improvement throughout treatment.

Figure 2 shows the clinical responses to secukinumab over time. The upper panel presents vertical bar graphs comparing (A) the cumulative prednisone doses during the 5 months before and after treatment and (B) the frequency of ENL episodes over the same period. The lower panel shows the individual trajectories of prednisone dose reduction after treatment initiation. The mean cumulative prednisone dose decreased from 279.0 ± 121.3 mg to 223.0 ± 59.7 mg after treatment. This comparison was assessed using a paired t-test, which did not show a statistically significant difference (*p*= 0.1764; 95% confidence interval [CI]: −38.79 to 150.8). In contrast, the mean frequency of ENL episodes significantly declined from 3.0 ± 0.7 to 0.6 ± 0.5 after secukinumab initiation (*p*= 0.0039; 95% CI: 1.290-3.510), also evaluated by paired t-test. The detailed numerical data are summarized in Table 2.

**FIGURE 2:**
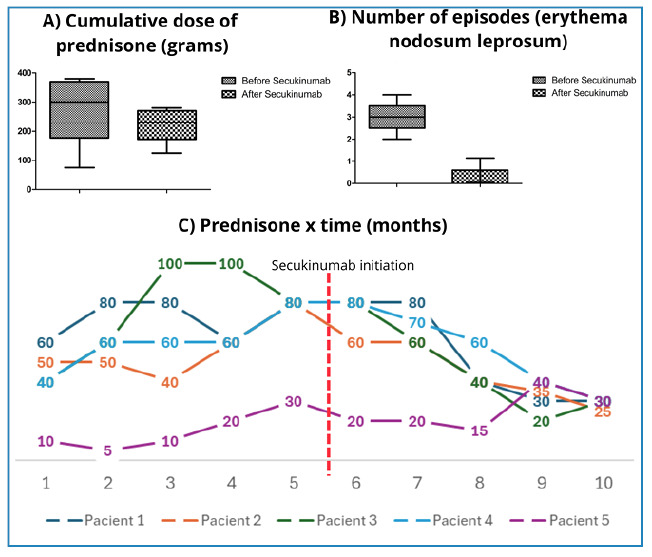
Box & whiskers graphs + Prednisone dose (mg) over time (months). **(A):** A vertical bar graph comparing the cumulative prednisone doses before and after secukinumab treatment initiation. Paired t-test comparing the accumulated dose between Column A and Column B showed no statistically significant difference (*p* = 0.1764). **(B):** A vertical bar graph comparing the median frequency of erythema nodosum leprosum (ENL) episodes before and after secukinumab initiation. Paired t-test comparing pre- and post-treatment with secukinumab showed a statistically significant difference between the groups (*p* = 0.0039). **(C):** Evolution of prednisone doses over time (in months) in the five patients treated with secukinumab. Each line represents the dose variation in a specific patient before and after secukinumab treatment initiation (indicated by the red dotted vertical line at month 6). A general trend of progressive dose reduction is observed after the start of therapy.

**TABLE 2:** Cumulative prednisone doses and ENL episode frequency before and after secukinumab treatment in patients with erythema nodosum leprosum.

Patient ID	Cumulative prednisone dose - 5 months before (mg)	Cumulative prednisone dose - 5 months after (mg)	Frequency of ENL episodes - 5 months before	Frequency of ENL episodes - 5 months after
**1**	360	260	4.0	0.0
**2**	280	220	3.0	1.0
**3**	380	230	2.0	0.0
**4**	300	280	3.0	1.0
**5**	75	125	3.0	1.0
**Mean ± SD**	279.0 ± 121.3	223.0 ± 59.7	3.0 ± 0.7	0.6 ± 0.5

**ENL:** erythema nodosum leprosum; **SD:** standard deviation. **Legend:** Cumulative prednisone doses (mg) and frequency of ENL episodes during the 5 months before and after secukinumab treatment in five patients with refractory ENL. Data are presented as individual values and as means ± SD.

No adverse events related to secukinumab were reported. Patient 2, who had the longest follow-up (15 months), maintained a favorable response and continued to progressively reduce corticosteroid use.

The pathogenesis of ENL involves a complex interplay of immune mechanisms, including type III hypersensitivity reactions, immune complex deposition, and neutrophil activation. The exact immunological profile is poorly understood and may contribute to variable responses to therapy^8^. However, emerging evidence suggests that cytokines, particularly TNF-α and specific interleukins, play central roles in disease activity^8^ ^,^ ^9^.

TNF has long been recognized as a key cytokine in ENL, with previous studies showing the efficacy of anti-TNF agents, such as infliximab and etanercept, in reducing disease severity. However, given the critical role of TNF in the host defense against other granulomatous infections, such as tuberculosis, which is highly prevalent in regions where leprosy is endemic, alternative therapeutic options should be considered to reduce the risk of mycobacterial reactivation^3^ ^,^ ^10^ ^,^ ^11^.

Recent evidence has highlighted the role of IL-17 in both major types of leprosy reactions, and IL-17-targeting biologics are emerging as lower-risk therapeutic options for these conditions. IL-17 can modulate neutrophil recruitment and tissue inflammation in leprosy reactions^6^ ^,^ ^7^. IL-17-producing γδ T cells have been shown to be significantly elevated in both ENL and reversal reactions, suggesting their involvement in the shared immunopathogenesis of these conditions^12^. Moreover, secukinumab was reported to modulate not only IL-17A but also Th1-associated cytokines such as interferon (IFN)-γ and TNF-α in a case of refractory neuritis, with marked clinical improvement^7^. These findings suggest that the IL-17 blockade may serve as a unified therapeutic strategy against different types of reactional states in leprosy.

Traditional treatments for ENL such as systemic corticosteroids and thalidomide are associated with significant toxicity, particularly when administered chronically. Corticosteroids can lead to a wide range of adverse effects, including metabolic complications (e.g., diabetes mellitus, weight gain), osteoporosis with a risk of pathological fractures, psychiatric disturbances, adrenal suppression, and increased susceptibility to serious infections. Despite its immunomodulatory efficacy, the use of thalidomide is limited by dose-dependent peripheral neuropathy, high teratogenic risk, sedation, and thromboembolic events that often require additional prophylaxis. These safety concerns underscore the need for alternative therapies for refractory cases^2^ ^,^ ^4^.

In contrast, secukinumab has shown a favorable safety profile in multiple clinical trials, with most adverse events being mild and self-limited, such as nasopharyngitis and mucocutaneous candidiasis^13^. In our study, no adverse events were reported, reinforcing its potential as a safer corticosteroid-sparing agent for ENL management.

Although other emerging therapies such as phosphodiesterase-4 inhibitors and ustekinumab have shown promising results in ENL, this report specifically addresses the use of an IL-17 inhibitor in its management^5^ ^,^ ^14^. Despite limitations, including the small sample size and retrospective design, our findings offer valuable insights into the potential role of IL-17 blockade in refractory ENL. Secukinumab appears to be a promising steroid-sparing option with favorable clinical responses and no observed adverse events. Further studies with larger cohorts and controlled designs are warranted to confirm these preliminary results and better define its long-term safety and efficacy in clinical practice.

## Data Availability

The data underlying this article will be shared upon reasonable request to the corresponding author.
